# Synchronous peritoneal metastases from lung cancer: incidence, associated factors, treatment and survival: a Dutch population-based study

**DOI:** 10.1007/s10585-021-10085-z

**Published:** 2021-03-18

**Authors:** Robin J. Lurvink, Anouk Rijken, Checca Bakkers, Mieke J. Aarts, Peter W. A. Kunst, Ben E. van de Borne, Felice N. van Erning, Ignace H. J. T. de Hingh

**Affiliations:** 1 Department of Surgery, Catharina Hospital, PO Box 1350, 5602 ZA Eindhoven, The Netherlands; 2 Department of Research and Development, Netherlands Comprehensive Cancer Organisation (IKNL), Utrecht, The Netherlands; 3 Department of Respiratory Medicine, Onze Lieve Vrouwe Gasthuis, Amsterdam, The Netherlands; 4 Department of Pulmonology, Catharina Hospital, Eindhoven, The Netherlands; 5 GROW – School for Oncology and Developmental Biology, Maastricht University, Maastricht, The Netherlands

**Keywords:** Peritoneal neoplasms, Lung neoplasms, Real world data, Neoplasm metastasis, Epidemiology

## Abstract

Peritoneal metastases (PM) from lung cancer are rare and it is unknown how they affect the prognosis of patients with lung cancer. This population-based study aimed to assess the incidence, associated factors, treatment and prognosis of PM from lung cancer. Data from the Netherlands Cancer Registry were used. All patients diagnosed with lung cancer between 2008 and 2018 were included. Logistic regression analysis was performed to identify factors associated with the presence of PM. Cox regression analysis was performed to identify factors associated with the overall survival (OS) of patients with PM. Between 2008 and 2018, 129,651 patients were diagnosed with lung cancer, of whom 2533 (2.0%) patients were diagnosed with PM. The European Standardized Rate of PM increased significantly from 0.6 in 2008 to 1.4 in 2018 (p < 0.001). Age between 50 and 74 years, T3–4 tumour stage, N2–3 nodal stage, tumour morphology of a small cell lung cancer or adenocarcinoma, and the presence of systemic metastases were associated with the presence of PM. The median OS of patients with PM was 2.5 months. Older age, male sex, T3–4 tumour stage, N2–3 nodal stage, not receiving systemic treatment, and the presence of systemic metastases were associated with a worse OS. Synchronous PM were diagnosed in 2.0% of patients with lung cancer and resulted in a very poor survival.

## Introduction

Lung cancer is the most common type of cancer worldwide, annually affecting more than 400,000 people in Europe alone [1]. Since half of the patients with lung cancer are simultaneously diagnosed with metastatic disease, the prognosis is generally poor, making lung cancer the leading cause of cancer-related death in Europe [1–4]. Despite the frequent encounter of systemic metastases, peritoneal metastases are rare and little is known about their incidence and how they affect survival. Available literature is limited to case reports and one population-based study focusing on peritoneal metastases from multiple extra-abdominal primary tumours [5–8]. The latter used the National Cancer Registry Ireland to identify 139 patients with peritoneal metastases from lung cancer.

Abdominal cancers have a higher tendency for peritoneal spread, affecting approximately 10% of these patients [9–12]. Although cytoreductive surgery with or without hyperthermic intraperitoneal chemotherapy is a treatment option for selected patients with peritoneal metastases from several abdominal cancers [9, 13], it is not available for patients with peritoneal metastases from extra-abdominal cancers, such as lung cancer. As a first step to guide future therapeutic research, the incidence of and associated factors for peritoneal metastases, as well as current treatment options and survival outcomes, should be explored.

Therefore, the aim of this study was to investigate the incidence of synchronous peritoneal metastases from lung cancer in a Dutch population-based cohort and to describe the characteristics, associated factors, treatment strategies and survival outcomes of these patients.

## Materials and methods

Data from the Netherlands Cancer Registry (NCR) were used [14]. The NCR registers all newly diagnosed cancers, and specifically trained data managers of the NCR obtain patient, tumour and treatment characteristics from the medical records. The topography and morphology of primary tumours and synchronous metastatic sites were recorded according to the International Classification of Diseases for Oncology [15, 16]. After the initial registration, the follow-up consists of a yearly evaluation of the vital status. All data are anonymized. No ethics approval was required for this study according to the Central Committee on Research involving Human Subjects in The Hague, the Netherlands. The privacy review board of the NCR approved the study.

All patients diagnosed with lung cancer between 1 January 2008 and 31 December 2018 were included in this study. Primary tumour morphologies according to the ICD-O [15] were divided in small cell lung cancer (SCLC; 8041–8045) and non-small cell lung cancer (NSCLC). NSCLC was subdivided into (1) squamous cell carcinoma (8070–8076, 8078, 8083, 8084, 8094), (2) adenocarcinoma (8140, 8144, 8250–8255, 8480, 8481, 8490, 8570, 8572, 8573), and (3) other (8001, 8002, 8010, 8012–8014, 8020, 8021, 8046, 8244, 8246, 8560, 8574). Other tumour morphologies, such as mesotheliomas and carcinoid tumours, were excluded. In case of multiple primary lung tumours in one patient, only the firstly diagnosed tumour was included. If multiple tumours were simultaneously diagnosed, the tumour with the highest stage was included.

The following metastatic sites were considered as peritoneal metastases: C16.0–C16.3, C16.5, C16.6, C16.8, C16.9, C17.0–C17.3, C17.8, C17.9, C18.0–C18.4, C18.6–C18.9, C19.9, C20.9, C21.8, C23.9, C26.9, C48.0–C48.2, C48.8, C49.4, C49.5, C52.9, C53.9, C54.0–C54.3, C54.8, C54.9, C55.9, C56.9, C57.0–C57.4, C57.8, C66.9, C67.0, C67.1, C67.4, C67.8, C67.9, C76.2, C76.3. All other metastatic sites were considered as systemic metastases.

The Tumour-Node-Metastasis (TNM) system was used to classify tumour characteristics. From 2008 to 2009, the sixth TNM edition was used; from 2010 to 2016, the seventh TNM edition was used; and from 2017 to 2018, the eighth TNM edition was used;

Patients were subcategorized into four groups: (1) patients with lung cancer without synchronous metastases, (2) patients with lung cancer and synchronous systemic metastases, (3) patients with lung cancer and synchronous peritoneal metastases, and (4) patients with lung cancer and both synchronous systemic and peritoneal metastases.

Treatment regimens were categorized as follows: (1) best supportive care only; (2) local treatment (comprising surgery and/or radiotherapy); and (3) systemic treatment (comprising chemotherapy and/or immunotherapy and/or targeted therapy).

The vital status was assessed on 31 January 2020 by linking the NCR to the Municipal Administrative Database, which comprises the vital status and date of death of all inhabitants of the Netherlands.

### Statistical analysis

Incidence rates of peritoneal metastases were calculated as the number of new patients per 100,000 inhabitants per year and were age standardized using both the European Standardized Rate (ESR) and the revised ESR [17]. The latter is the most up-to-date method for calculating incidence rates, but the former has frequently been used in previous studies, facilitating comparison to available literature. Trends over time were assessed through the Estimated Annual Percent Change (EAPC). Categorical variables were represented as n (%) and compared between the four groups with the Chi-square test. Continuous variables were represented as mean ± standard deviation and compared between the four groups with the One-Way Anova test. Univariable logistic regression analysis was performed to identify characteristics associated with the presence of synchronous peritoneal metastases (p < 0.10) which were subsequently combined in a multivariable logistic regression model. Overall survival (OS) of patients with peritoneal metastases was presented with the Kaplan Meier method and compared with the Log-rank test (solitary peritoneal metastases vs. peritoneal and systemic metastases). OS was defined as the time from diagnosis of the primary tumour until death or last follow-up date (31 January 2020). Univariable cox regression analysis was performed in all patients with peritoneal metastases to identify characteristics associated with a worse OS (p < 0.10) which were subsequently combined in a multivariable cox regression model. All statistical analyses were performed with SAS 9.4 (SAS Institute, North Carolina, United States). A p-value p < 0.05 was considered statistically significant.

## Results

The final study population comprised 129,651 patients with lung cancer. Within this group, 62,890 (48.5%) patients did not have synchronous systemic metastatic disease, 64,228 (49.5%) patients had synchronous systemic metastases only, 326 (0.3%) patients had synchronous peritoneal metastases only, and 2207 (1.7%) patients had both synchronous systemic and peritoneal metastases. Thus, a total of 2533 (2.0%) patients with lung cancer were diagnosed with synchronous peritoneal metastases.

Table 1 contains the baseline characteristics of (1) patients with lung cancer without synchronous metastases, (2) patients with lung cancer and synchronous systemic metastases, (3) patients with lung cancer and synchronous peritoneal metastases, and (4) patients with lung cancer and both synchronous systemic and peritoneal metastases. Patients with metastatic disease more often had a SCLC tumour histology than patients without metastatic disease (13–21% vs. 10%, respectively). This difference was more pronounced for patients with systemic metastases (19%) and patients with systemic and peritoneal metastases (21%) than for patients with solitary peritoneal metastases (13%). A similar trend was observed for tumour stage, nodal stage, and World Health Organization (WHO) performance status: patients with metastatic disease were more likely to have a T3–4 tumour stage or N2–3 nodal stage or WHO performance status 2–4 than patients without systemic metastatic disease (T3–4 tumour stage: 54–58% vs. 36%, respectively [p < 0.001]; N2–3 nodal stage: 60–77% vs. 40%, respectively [p < 0.001]; WHO performance status 2–4: 23–43% vs. 20%, respectively [p < 0.001]).

**Table 1:** Baseline characteristics of patients with lung cancer

	No synchronous systemic metastases N = 62,890		Synchronous systemic metastases N = 64,228		Synchronous peritoneal metastases N = 326		Synchronous systemic and peritoneal metastases N = 2207		P value
Sex									< 0.001
Male	37,466 (60)		37,269 (58)		181 (55)		1302 (59)		
Female	25,424 (40)		26,959 (42)		145 (45)		905 (41)		
Age at diagnosis									<** 0**.**001**
Years	69 ± 10		68 ± 11		67 ± 11		67 ± 10		
Histology									<** 0**.**001**
SCLC	6026 (10)		12,261 (19)		42 (13)		458 (21)		
NSCLC									
Adenocarcinoma	20,183 (32)		27,872 (43)		159 (49)		963 (44)		
Squamous cell carcinoma	19,532 (31)		8173 (13)		47 (14)		211 (9)		
Other	17,149 (27)		15,922 (25)		78 (24)		575 (26)		
Tumour stage									<** 0**.001*
T0–2	36,549 (58)		21,760 (34)		99 (30)		674 (31)		
T3–4	22,680 (36)		34,887 (54)		180 (55)		1288 (58)		
Missing data	3661 (6)		7581 (12)		47 (14)		245 (11)		
Nodal stage									<** 0**.001*
N0–1	34,374 (55)		12,055 (19)		92 (28)		344 (16)		
N2	16,829 (27)		23,977 (37)		115 (35)		827 (37)		
N3	8131 (13)		22,889 (36)		81 (25)		892 (40)		
Missing data	3556 (6)		5307 (8)		38 (12)		144 (7)		
WHO performance									<** 0**.001*
WHO 0–1	12,609 (20)		10,151 (16)		68 (21)		370 (17)		
WHO 2–4	3189 (5)		5223 (8)		26 (8)		272 (12)		
Missing data	47,092 (75)		48,854 (76)		232 (72)		1565 (71)		

p values < 0.05 are in bold

All values are n (%) or mean ± standard deviation

*NSCLC* Non-small cell lung cancer, *PM* peritoneal metastases, *SCLC* small cell lung cancer, *WHO* World Health Organization

*Missing values were excluded from chi-square analyses

**Fig. 1:**
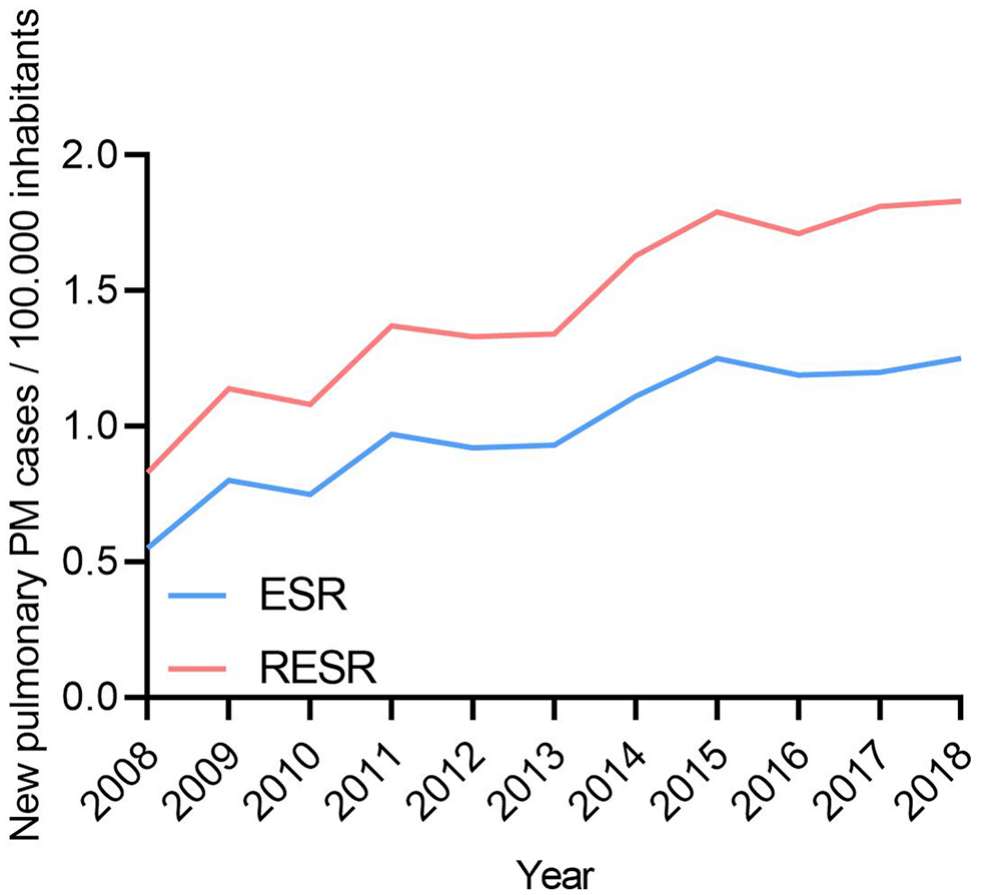
European Standardized Rate of pulmonary peritoneal metastases over time. *ESR* European Standardized Rate; *PM* peritoneal metastases; *RESR* Revised European Standardized Rate

Figure 1 presents the ESR and the revised ESR of lung cancer with peritoneal metastases (with or without systemic metastases) from 2008 to 2018. The ESR significantly increased from 0.6 in 2008 to 1.3 in 2018 (EAPC of 7.3%, p < 0.001), as well as the revised ESR, which increased from 0.8 in 2008 to 1.8 in 2018 (EAPC of 7.4%, p < 0.001).

**Fig. 2:**
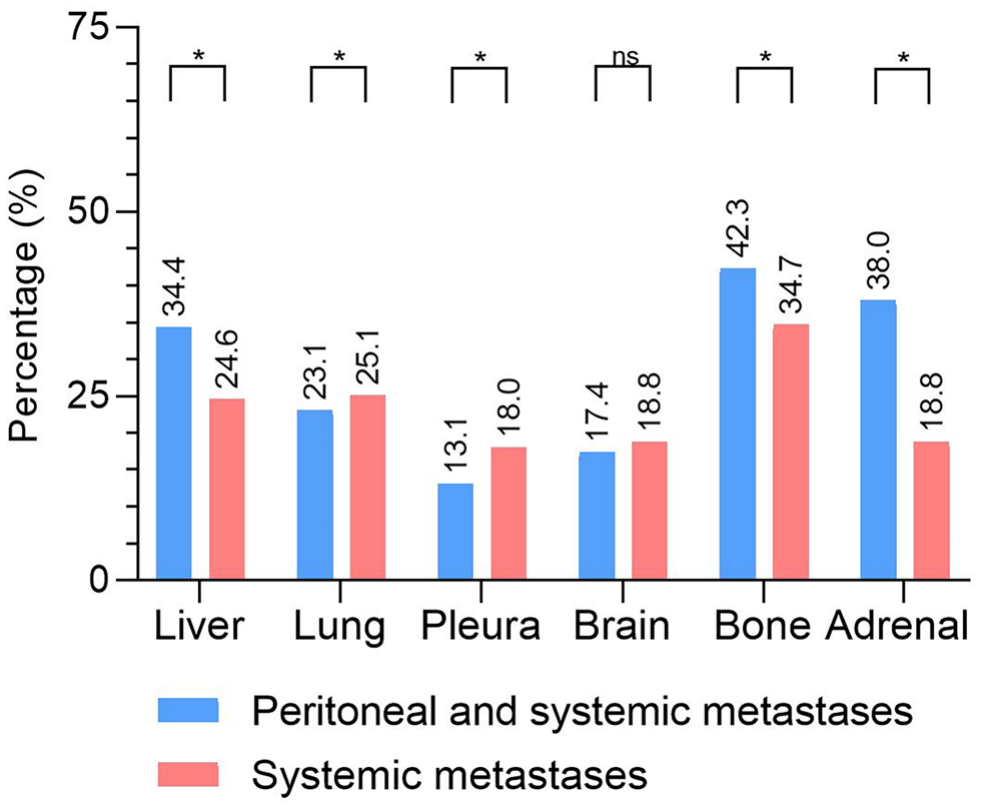
Patterns of synchronous systemic metastases. *NS* not statistically significant; *statistically significant

Figure 2 shows an overview of patterns of synchronous systemic metastases, stratified for patients with lung cancer and synchronous systemic metastases and for patients with lung cancer and both synchronous systemic and peritoneal metastases. Remarkably, patients with both synchronous systemic and peritoneal metastases more often had systemic metastases located in the liver, bones, and adrenal glands, whereas patients with synchronous systemic metastases more often had systemic metastases located in the lungs and pleura. Brain metastases were equally diagnosed in both groups.

### Factors associated with peritoneal metastases

Table 2 presents the results of univariable and multivariable logistic regression analyses. These showed that patients aged 50–74 had a higher odds (OR 1.17 [95% Confidence Interval (CI) 1.08–1.27]) of having synchronous peritoneal metastases compared to patients aged ≥ 75 years. Furthermore, tumour histology of a small cell lung cancer (OR 1.54 [95% CI 1.32–1.80]) or adenocarcinoma (OR 1.58 [95% CI 1.37–1.82]), a T3–4 tumour stage (OR 1.30 [95% CI 1.19–1.43]), an N2–3 nodal stage (N2: OR 1.40 [95% CI 1.24–1.57]; N3: OR 1.53 [95% CI 1.35–1.72]), a World Health Organization (WHO) performance status of 2-4 (OR 1.45 [95% CI 1.25–1.69]), and the presence of synchronous systemic metastases (OR 5.02 [95% CI 4.44–5.69]), were significantly associated with the presence of synchronous peritoneal metastases.

**Table 2:** Logistic regression analysis for the presence of synchronous peritoneal metastases in patients with lung cancer

	Peritoneal metastases	Univariate logistic regression analysis			Multivariate logistic regression analysis		
	n (%)	OR	95% CI	P value	OR	95% CI	P value
Age at diagnosis				<** 0**.**001**			
< 50 years	107 (2)	1.18	0.97–1.45		1.00	0.81–1.22	0.977
50–74 years	1348 (2)	1.26	1.16–1.37		**1**.**17**	**1**.**08**–**1**.**27**	<** 0**.**001**
≥ 75 years	1078 (2)	Ref	Ref		Ref	Ref	Ref
Sex				0.804			
Male	1483 (2)	Ref	Ref		–	–	–
Female	1050 (2)	1.01	0.93–1.09		–	–	–
Tumour morphology				<** 0**.**001**			
SCLC	500 (3)	2.94	2.52–3.42		**1**.**54**	**1**.**32**–**1**.**80**	<** 0**.**001**
NSCLC							
Adenocarcinoma	1122 (2)	2.51	2.19–2.87		**1**.**58**	**1**.**37**–**1**.**82**	<** 0**.**001**
Squamous cell carcinoma	258 (1)	Ref	Ref		Ref	Ref	Ref
Other	653 (2)	2.12	1.83–2.45		**1**.**56**	**1**.**35**–**1**.**81**	<** 0**.**001**
Tumour stage				<** 0**.**001**			
T0–2	773 (1)	Ref	Ref		Ref	Ref	Ref
T3–4	1468 (3)	1.92	1.76–2.10		**1**.**30**	**1**.**19**–**1**.**43**	<** 0**.**001**
Missing data	292 (3)	1.96	1.71–2.25		1.28	1.10–1.48	0.001
Nodal stage				<** 0**.**001**			
N0–1	436 (1)	Ref	Ref		Ref	Ref	Ref
N2	942 (2)	2.46	2.19–2.76		**1**.**40**	**1**.**24**–**1**.**57**	<** 0**.**001**
N3	973 (3)	3.34	2.98–3.74		**1**.**53**	**1**.**35**–**1**.**72**	<** 0**.**001**
Missing data	182 (2)	2.19	1.84–2.60		1.22	1.02–1.48	0.034
WHO performance				<** 0**.**001**			
WHO 0–1	438 (2)	Ref	Ref		Ref	Ref	Ref
WHO 2–4	298 (3)	1.84	1.59–2.14		**1**.**45**	**1**.**25**–**1**.**69**	<** 0**.**001**
Missing data	1797 (2)	0.97	0.88–1.08		0.90	0.81–1.01	0.058
Synchronous systemic metastases				<** 0**.**001**			
No	326 (1)	Ref	Ref		Ref	Ref	Ref
Yes	2207 (3)	6.63	5.90–7.45		**5**.**02**	**4**.**44**–**5**.**69**	<** 0**.**001**

p values < 0.05 are in bold

*CI* confidence interval, *OR* odds ratio, *NSCLC* non-small cell lung cancer, *SCLC* small cell lung cancer, *WHO* World Health Organization

### Treatment of peritoneal metastases

The majority of patients with synchronous peritoneal metastases only received best supportive care (n = 1754, 69%). The remaining patients received systemic treatment (n = 354, 14%), local treatment (surgery and/or radiotherapy; n = 189, 7%), or both (n = 236, 9%).

Patients with solitary synchronous peritoneal metastases more often only received best supportive care than patients with peritoneal and systemic metastases (78% vs. 68%, respectively [p < 0.001]). Systemic treatment was administered to 19% of patients with solitary synchronous peritoneal metastases and to 24% of patients with synchronous peritoneal and systemic metastases (p = 0.051). Patients with solitary synchronous peritoneal metastases less often received local treatment than patients with synchronous peritoneal and systemic metastases (8% vs. 18%, p < 0.001).

### Survival of patients with metastatic lung cancer

The median OS of all patients with synchronous peritoneal metastases was 2.5 months (interquartile range [IQR] 1.0–6.6), and the 1- and 2-year survival rates were 12.0% and 4.0%, respectively.

**Fig. 3:**
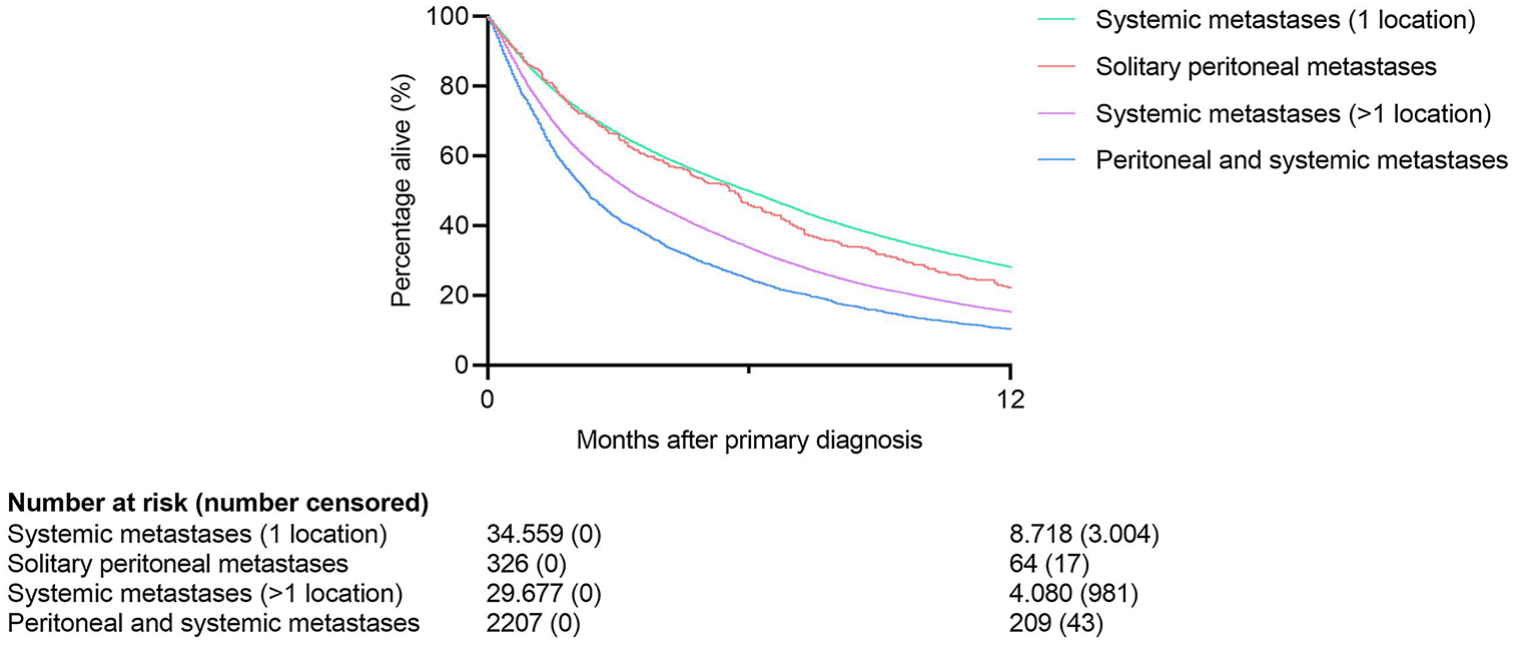
Overall survival of patients with metastatic lung cancer. *PM* peritoneal metastases

Patients with solitary peritoneal metastases had a median OS of 5.6 months (IQR 1.9–11.0) and a 1- and 2-year survival rate of 22.1% and 10.5%, respectively (Fig. 3). Patients with systemic metastases in one location had a median OS of 6.0 months (IQR 1.9–13.5) and a 1- and 2-year survival rate of 28.2% and 12.5%, respectively (Fig. 3). The survival of patients with solitary peritoneal metastases was not significantly different from patients with systemic metastases in one location (p = 0.199).

Patients with peritoneal and systemic metastases had a median OS of 2.3 months (IQR 1.0–6.0) and a 1- and 2-year survival rate of 10.4% and 3.0%, respectively (Fig. 3). Patients with systemic metastases in more than one location (but not including peritoneal) had a median OS of 3.3 months (IQR 1.2–8.1) and a 1- and 2-year survival rate of 15.3% and 5.6%, respectively (Fig. 3). The survival of patients with peritoneal and systemic metastases was significantly worse than patients with systemic metastases in more than one location (p < 0.001).

Among patients with synchronous peritoneal metastases, multivariable cox regression analysis showed that younger age (< 50 years: HR 0.77 [95% CI 0.63–0.95]); 50–74 years: HR 0.85 [95% CI 0.78–0.93]), female sex (HR 0.89 [95% CI 0.82–0.97]), and systemic treatment (HR 0.54 [95% CI 0.49–0.60]) were associated with a better OS. A T3–4 tumour stage (HR 1.19 [95% CI 1.09–1.31]), an N2–3 nodal stage (N2: HR 1.29 [95% CI 1.14–1.45]; N3: HR 1.31 [95% CI 1.17–1.48]), a WHO performance status 2–4 (HR 2.33 [95% CI 1.99–2.71]), and the presence of synchronous systemic metastases (HR 1.72 [95% CI 1.52–1.95]) were associated with a worse OS (Table 3).

**Table 3:** Cox regression analysis for the survival of patients with lung cancer with synchronous peritoneal metastases

	Median survival	Univariate Cox regression analysis			Multivariate Cox regression analysis		
	(months)	HR	95 %CI	P value	HR	95 %CI	P value
Age at diagnosis				<** 0**.**001**			
< 50 years	3.7	0.68	0.55–0.83		**0**.**77**	**0**.**63**–**0**.**95**	**0**.**015**
50–74 years	3.0	0.77	0.71–0.83		**0**.**85**	**0**.**78**–**0**.**93**	<** 0**.**001**
≥ 75 years	2.0	Ref	Ref		Ref	Ref	Ref
Sex				<** 0**.**001**			
Male	2.3	Ref	Ref		Ref	Ref	Ref
Female	2.8	0.56	0.79–0.93		**0**.**89**	**0**.**82**–**0**.**97**	**0**.**006**
Tumour morphology				<** 0**.**001**			
SCLC	3.5	0.94	0.81–1.09		0.96	0.82–1.12	0.566
NSCLC							
Adenocarcinoma	2.7	0.92	0.81–1.06		0.99	0.86–1.14	0.898
Squamous cell carcinoma	2.7	Ref	Ref		Ref	Ref	Ref
Other	1.9	1.25	1.08–1.44		**1**.**21**	**1**.**05**–**1**.**40**	**0**.**011**
Tumour stage				<** 0**.**001**			
T0–2	3.2	Ref	Ref		Ref	Ref	Ref
T3–4	2.4	1.20	1.10–1.31		**1**.**19**	**1**.**09**–**1**.**31**	<** 0**.**001**
Missing data	1.7	1.47	1.28–1.68		1.28	1.11–1.48	< 0.001
Nodal stage				<** 0**.**001**			
N0–1	3.7	Ref	Ref		Ref	Ref	Ref
N2	2.4	1.32	1.17–1.48		**1**.**29**	**1**.**14**–**1**.**45**	<** 0**.**001**
N3	2.4	1.36	1.21–1.52		**1**.**31**	**1**.**17**–**1**.**48**	<** 0**.**001**
Missing data	1.4	1.92	1.61–2.29		1.77	1.47–2.12	< 0.001
WHO performance				<** 0**.**001**			
WHO 0–1	5.2	Ref	Ref		Ref	Ref	Ref
WHO 2–4	1.5	2.45	2.10–2.85		**2**.**33**	**1**.**99**–**2**.**71**	<** 0**.**001**
Missing data	2.3	1.66	1.49–1.85		**1**.**72**	**1**.**54**–**1**.**92**	<** 0**.**001**
Systemic treatment				<** 0**.**001**			
No	1.9	Ref	Ref		Ref	Ref	Ref
Yes	5.6	0.58	0.53–0.64		**0**.**54**	**0**.**49**–**0**.**60**	<** 0**.**001**
Local treatment				<** 0**.**001**			
No	2.3	Ref	Ref		Ref	Ref	Ref
Yes	3.7	0.83	0.75–0.92		1.02	0.91–1.14	0.748
Synchronous systemic metastases				<** 0**.**001**			
No	5.6	Ref	Ref		Ref	Ref	Ref
Yes	2.3	1.66	1.47–1.87		**1**.**72**	**1**.**52**–**1**.**95**	<** 0**.**001**

p values < 0.05 are in bold

*CI* confidence interval, *HR* hazard ratio, *NSCLC* non-small cell lung cancer, *SCLC* small cell lung cancer

## Discussion

This study aimed to provide an overview of the incidence, associated factors, treatment, and survival of patients with lung cancer with synchronous peritoneal metastases. Synchronous peritoneal metastases were found in 2.0% of patients with lung cancer. Most patients with synchronous peritoneal metastases also had synchronous systemic metastases. The incidence of synchronous peritoneal metastases in patients with lung cancer increased over time. Younger age, a poorer WHO performance status, SCLC or adenocarcinoma tumour histology, and advanced disease (both T, N, and M stage) were associated with the presence of synchronous peritoneal metastases. The median OS of all patients with synchronous peritoneal metastases was 2.5 months, and an older age, male sex, a poorer WHO performance status, advanced disease (both T, N and M stage), and not receiving systemic treatment were associated with a worse OS.

An Irish population-based cohort identified that 0.4% of patients with lung cancer were diagnosed with synchronous or metachronous peritoneal metastases [7]. They reported a much lower incidence of peritoneal metastases than the current study. This is most likely related to the improvement and increased use of diagnostic modalities [18], such as (FDG-PET) computed tomography, and the increasing knowledge and awareness of peritoneal metastases over time. The analysis from Flanagan et al. was performed with patients diagnosed between 1999 and 2012, whereas the current study was performed with patients diagnosed between 2008 and 2018. Three other studies also described small cohorts of patients with lung cancer with peritoneal metastases, which summed up to a total of 66 patients with peritoneal metastases. In these studies, the incidence of peritoneal metastases ranged from 0.8 to 1.2% [6, 19, 20]. These studies reported on patients diagnosed with lung cancer and peritoneal metastases between 1990 and 2012.

Even so, the reported incidence of peritoneal metastases in the current study is likely to be an underestimation of the true incidence: the current cohort did not include metachronous peritoneal metastases, whereas Flanagan et al. reported that a third of the patients with lung cancer with peritoneal metastases had a metachronous onset of peritoneal metastases. Furthermore, peritoneal metastases are not easily detected on abdominal CT-scans, nor is a diagnostic laparoscopy or laparotomy routinely performed in patients with lung cancer, which has probably resulted in missed diagnoses of synchronous peritoneal metastases. Therefore, the currently reported incidence of peritoneal metastases from lung cancer is likely an underestimation. This is reflected by the much higher incidence rates of peritoneal metastases from autopsy studies, where peritoneal metastases are found in 2.7–16.0% of patients with lung cancer [21–23].

The current study showed that patients with lung cancer with peritoneal metastases have a poor prognosis. This is comparable to the median OS of 2.0–2.8 months reported in other cohorts [6, 7, 19, 20]. However, in contrast to these studies, the current study showed that patients with solitary peritoneal metastases have a more favourable OS than patients with peritoneal and systemic metastases. Hypothetically, this might be related to differences in the chosen treatment. However, this study found that systemic therapy was equally offered to both patients with solitary peritoneal metastases and to patients with peritoneal and systemic metastases.

In the current study, 69% of patients with peritoneal metastases did not receive either systemic or local treatment. This remarkably high number could partially be explained given that a quarter of the patients had already died during the first month after diagnosis, possibly withholding them from starting with any treatment. Nevertheless, the Irish cohort also reported on a high percentage of patients (48%) who did not receive tumour-directed treatment. This possibly reflects the extremely poor condition of patients with peritoneal metastases from lung cancer, given that 34–50% of patients with lung cancer are considered to have an Eastern Cooperative Oncology Group performance scale ≥ 2, severely limiting their treatment options [24–26]. Since these analyses were performed on general lung cancer populations, it is likely that the performance status of patients with metastatic lung cancer is even worse.

Finally, several factors were identified which were associated with a higher incidence of peritoneal metastases: younger age, a higher tumour and nodal stage, a poorer WHO performance status, and the presence of synchronous systemic metastases. The higher incidence in patients of younger age could be biased, since younger patients with good clinical condition are more likely to undergo intensive diagnostic work-up and treatment, increasing the chance of finding peritoneal metastases. A higher tumour and nodal stage and the presence of synchronous systemic metastases were also identified by other studies to be associated with a higher odds of peritoneal metastases from other primary tumours, such as gastric, ovarian, and colorectal cancer [11, 12, 27].

Also, several factors were associated with a worse OS, such as older age, male gender, not receiving systemic treatment, the presence of synchronous systemic metastases, and higher tumour and nodal stages. Although these factors are generally associated with a worse OS in patients with lung cancer, regardless of peritoneal metastases, these factors were also identified to be associated with a worse OS in patients with peritoneal metastases from colon and pancreatic cancer [28, 29].

This study also has some limitations. First, peritoneal metastases are not easily detected on imaging, and diagnostic laparoscopy or laparotomy is infrequently performed in patients with lung cancer. Thus, the diagnosis of peritoneal metastases in patients with lung cancer is most likely a coincidental finding, hence the currently reported incidence is probably an underestimation of the actual incidence of peritoneal metastases in patients with lung cancer. Furthermore, the presence of comorbidities was not available and therefore not included in logistic and cox regression analyses. Instead, the performance status was analyzed but this was only registered from 2015 onwards, resulting in missing data in a proportion of the patients.

The current study is, to the best knowledge of the authors, the largest population-based study to date to provide insight into the incidence, associated factors, treatment and prognosis of patients with lung cancer with peritoneal metastases. Also, the NCR is characterized by a high registration coverage of more than 95% of all diagnosed cancers and standardized registration [14].

## Conclusion

Synchronous peritoneal metastases are diagnosed in 2.0% of patients with lung cancer. Most patients did not receive treatment and survival is very poor.

## Abbreviations

CI: Confidence interval
CT: Computed tomography
EAPC: Estimated annual percent change
ESR: European Standardized Rate
HR: Hazard ratio
ICD-O: International Classification of Disease-Oncology
IQR: Interquartile range
NCR: Netherlands Cancer Registry
NSCLC: Non-small cell lung cancer
OR: Odds ratio
OS: Overall survival
PM: Peritoneal metastases
SCLC: Small cell lung cancer
TNM: Tumour node metastasis
